# Barriers and determinants of postpartum family planning uptake among postpartum women in Western Ethiopia: a facility-based cross-sectional study

**DOI:** 10.1186/s13690-022-00786-6

**Published:** 2022-01-12

**Authors:** Temesgen Tilahun, Tariku Tesfaye Bekuma, Motuma Getachew, Rut Oljira, Assefa Seme

**Affiliations:** 1 Department of Obstetrics & Gynecology, School of medicine, Institute of Health Sciences, Wollega University, Nekemte, Ethiopia; 2 Department of Public Health, Institute of Health Sciences, Wollega University, Nekemte, Ethiopia; 3 Department of Reproductive Health and Health Service Management, College of Health Sciences, Addis Ababa University, Addis Ababa, Ethiopia

**Keywords:** Post-partum, Family planning, Barriers, Western Ethiopia

## Abstract

**Background:**

Despite Ethiopia’s efforts to avail postpartum family planning (PPFP) services, the unmet need for family planning among postpartum women remains high. Therefore, this study is aimed to assess barriers and determinants of postpartum family-planning uptake among women visiting Maternal, Neonatal, and Child Health (MNCH) services in public health facilities of western Ethiopia.

**Methods:**

A facility-based cross-sectional study design with a quantitative method was conducted on 989 postpartum women in Western Ethiopia from September 1 to October 30, 2020. Data were collected through face-to-face interviews using pretested structured questionnaires, entered using EPI-INFO version 7.0, and analyzed by SPSS version 25. Descriptive analysis and logistic regressions were performed. The adjusted odds ratio with a 95% confidence interval was used and statistical significance was declared at *P*-value < 0.05.

**Result:**

In this study, 56.1% of participants had used PPFP in the last year. The most commonly used method was injectable (51.7%). Family planning use before the index pregnancy (AOR = 2.09;95%CI:1.29,3,41),counselling on PPFP during antenatal care and delivery (AOR = 4.89;95%CI:2.31,10.37),health facility delivery (AOR = 7.61;95%CI:4.36,13.28), skilled birth attendance (AOR = 4.99;95%CI:2.88,8.64),COVID-19 restrictions (AOR = 0.59;95%CI:0.39,0.90) were factors associated with PPFP utilization. Being breastfeeding and amenorrhea were major reasons for not using postpartum family planning.

**Conclusion:**

Post-partum family planning utilization among study participants was low. Given the associated factors, it is recommended that health facilities should make postpartum family planning one of their top priorities and focus on these factors to improve its utilization.

## Background

The postpartum period is a critical time to address the high unmet need of family planning and to reduce the risks of closely spaced pregnancies [1]. Contraception is particularly important during the postpartum period to deter terrible maternal, neonatal, and child morbidity and mortality. Since 2010, the World Health Organization (WHO) has been receiving an increasing number of requests from country programs for strategies to create or strengthen voluntary family planning programs for women in the first year after childbirth [2]. As many as 50–90% of women from 17 low- and middle-income countries (LMICs) report an unmet need for postpartum family planning (PPFP) [2]. Women frequently return to fertility before initiating contraception after delivery and do not necessarily understand the risk of pregnancy before the return of menses [3]. WHO recommends PPFP as a critical component of health care that has the potential to meet women’s desire for contraception and save millions of maternal and infant lives in low- and middle-income countries [4].

The uptake of postpartum family planning in sub-Saharan Africa remains low, especially with the lowest use observed in East Africa which varied from 10.3% in Ethiopia to 73.7% in Uganda [5]. Perceptions of low pregnancy risk due to breastfeeding and postpartum amenorrhea, fear of method-related side effects, and poor family planning counseling were factors associated with the low uptake of PPFP while women who received good PPFP counseling during antenatal and postnatal care were more likely to use PPFP [4, 5].

The Ethiopian Health Sector Development Program (HSDP) IV sets a goal of improving maternal health and increasing family planning coverage. However, the first year after birth is given less emphasis regarding contraceptive utilization [6]. In Ethiopia, nearly half of all non-first pregnancies occur less than 24 months following the preceding birth [7].

Studies have revealed that the first year following delivery is so complex and different from other times in a woman’s life cycle due to the additional burden to care for her infant and the series of emotional and physical changes [5–7].

The prevalence of contraceptive use among postpartum women varies from region to region in Ethiopia, as most women do not start taking contraceptives at the recommended time [7, 8]. Even those who use PPFP rely on traditional, mainly lactational amenorrhea (LAM) that might pose the risk of unintended pregnancy. Therefore, initiating appropriate contraception in the postpartum period is important to avoid negative health outcomes [8].

Few studies conducted in Ethiopia indicated that PPFP uptake is low with the most common determinants affecting uptake being sociodemographic characteristics, antenatal care (ANC) attendance, resumption to sexual activities, postnatal care (PNC) attendance, the return of menses, and duration after delivery [3, 9–11]. These studies tried to measure magnitude and factors that are positively associated with PPFP uptake and, none of these studies so far have well-addressed barriers to PPFP uptake.

In general, studies in Ethiopia were conducted in towns and might not represent postpartum family planning uptake and possible associated factors in a rural community. Governmental attention, health facilities’ concerns, and readiness, and health professional factors were not well investigated. Thus, the current study will address associated factors of postpartum family planning utilization in a rural part of Ethiopia.

## Methods

### Study setting and design

A cross-sectional quantitative study design was conducted in Western Ethiopia (Western Oromia and Benishangul Gumuz Regional State) public health facilities from September 1 to October 30, 2020. From Western Oromia, the zones included were East wollega, Horroguduru wollega, and west Wollega. Benishangu Gumuz Regional State is one of the 10 regional states in Ethiopia. It is adjacent to the Western part of Oromia regional state. Assosa is the capital city of this region and is located 670 km from the west of Addis Ababa. Assosa zone is included in this study. The study is conducted in five hospitals and ten health centers in western Oromia, and two hospitals and five health centers from Benishangu Gumuz Regional State.

### Study design

A cross-sectional study design with a quantitative method was conducted.

### Source population

The source population for this study was all postpartum mothers aged between 15 to 49 years who were living in the catchment population of the study facilities.

### Study population

The study population was postpartum mothers who have given birth in the last 12 months of the study period and visiting the selected study hospitals and health centers for any maternal, neonatal, and child health services.

### Eligibility criteria

#### Inclusion criteria

Postpartum women who gave birth in the last 12 months.

Postpartum mother who gave her consent to the study.

#### Exclusion criteria

Postpartum mothers who were severely sick and unable to talk were excluded from the study.

### Sample size determination

The single population proportion formula was used to calculate the sample size with the following assumption. The proportion of postpartum women who utilized postpartum family planning which was 48.11% was taken [10]. The marginal error of 4%, design effect 1.5, and 95% confidence level. After adding a 10% non-response rate, the final sample size was 990.

### Sampling techniques

In this study, the East Wollega zone, Horroguduru Wollega, and West wollega zones were included from western Oromia while the Assosa zone was included from Benishangul Gumuz. All types of hospitals and district-level health centers that are currently providing postpartum family planning services were considered in the study. The total list of hospitals and health centers in the study zones was obtained from zonal health departments of the study zones. Then, the study hospitals and health centers were selected by simple random sampling. The weekly average client load of the target age group in the MNCH department of study health facilities was taken from registry books and the respective sample size for each selected health facility was allocated proportionally to their MNCH department client flow. Finally, an eligible postpartum woman who had been selected by systematic random sampling was interviewed at an entry point.

### Study variables

#### Dependent variable

Postpartum family planning uptake.

#### Independent variables

socio-demographic characteristics, husband education and occupation, obstetric factors, family planning related factors, breast feeding, facility readiness, place of delivery, and COVID-19 pandemic restrictions.

### Data collection procedures

Data were collected by 25 data collectors who know Afan Oromo and Amharic. The quantitative data were collected through interviewer-administered interviews using adopted questionnaires from reviewed literature on postpartum family planning [3, 10–25]. Inorder to minimize potential bias, the authors used precise tools and well trained female BSC nurses,who were not working in the same facility/study area, for data collection.

### Data processing and analysis

The data were checked, entered, and cleaned using EPI-INFO version 7.0 and then exported to Statistical Package for Social Sciences (SPSS) software package version 25 for analysis. Using the odds ratio (OR) with a 95% limit of the confidence interval, the association of dependent and independent variables was identified, and their degree of association was computed. Potential confounding variables were controlled by using multiple logistic regression.

Descriptive statistics (like frequencies, percentages) were used to describe the study population in relation to dependent and independent variables. Results were presented in text, graph, charts, and tables.

### Data quality control

The training was given for the data collectors and supervisors on the objective of the study, how to conduct interviews. The data collection was regularly monitored daily for completeness. Data collection was done in the local language. Moreover, the field teams were provided the necessary personal protective equipment during the training, pretest, and actual data collection. As a precaution for COVID-19, the study participants were also provided face masks during the interview process.

## Results

### Socio-demographic characteristics of participants

In this study, 989 postpartum women were participated making a response rate of 99.89%. Six hundred thirty (63.70%) and 359(36.30%) of participants were from Western Oromia and Beneshangul Gumuz Regional States respectively. Regarding the age group of participants, the result indicated that more than two-third, 649 (65.62%) were those aged between 20 to 29 years. The majority, 599 (60.57%), of the participants were from rural areas. Six hundred eight nine (69.7%) of the participants were Oromo (69.67%) followed by Amhara 151 (15.3%)(Table 1).

**Table 1:** Sociodemographic characteristics of study participants, Western Ethiopia, 2020

Characteristics		Frequency	Percentage
**Region**	Oromia Regional State	630	63.7
	Benshangul Gumuz Regional State	359	36.3
**Age in years**	15 to 19	36	3.6
	20 to 24	235	23.7
	25 to 29	414	41.8
	30 to 34	209	21.1
	> 35	95	9.6
**Residence**	Urban	599	60.5
	Rural	390	39.4
**Religion**	Protestant	484	48.9
	Orthodox	245	24.7
	Muslim	234	23.6
	Adventist	15	1.5
	Catholic	6	0.6
	Wakefata	5	0.5
**Marital status**	Married	955	96.5
	Never married	17	1.7
	Others	17	1.7
**Ethnicity**	Oromo	689	69.6
	Amhara	151	15.2
	Berta	95	9.6
	Others	46	4.6
**Participant education**	Cannot read and write	189	19.1
	Can read and write	79	7.9
	Grade 1–8	255	25.7
	Grade 9–12	239	24.1
	College & above	227	22.9
Participant occupation	Farmer	376	38.0
	Government employee	309	31.2
	NGO employee	26	2.6
	Merchant	81	8.1
	Daily laborer	143	14.4
	Student	25	2.5
**Husband education**	Cannot read & write	110	2.5
	Read & write	80	11.1
	Grade 1 to 8	181	8.1
	Grade 9 to12	245	18.3
	College & above	344	24.7
**Husband occupation**	Farmer	405	40.9
	Government employee	309	31.2
	NGO employee	26	2.6
	Merchant	81	8.2
	Daily laborer	143	14.4
	Student	25	2.5

### Reproductive characteristics of study participants

In this study, the majority of study participants, 48.6%, were para 2 and 3. Most of the participants, 817 (82.6%), had ANC follow-up for the index pregnancy. From these, more than half, 459 (56.18%) had at least four antenatal care visits. One hundred thirty-six (13.8%) of study participants had a history of abortion. One hundred nine (11%) had ever encountered unintended pregnancy and 63 (6.40%) had a history of stillbirth**.** Three fourth, 744(75.2), of the study participants, had attended postnatal care. Among them, the majority 341(45.8%) had attended postnatal care from 2 to 6 weeks. Two hundred eight (37.6%) of participants attended postnatal care before the end of one week. More than 99% of these women attended postnatal care at health facilities. Eight hundred thirteen (82.2%) of the women are reported to be currently breastfeeding **(**Table 2**).**

**Table 2:** Maternal health service utilization among study participants, Western Ethiopia, 2020

Variables	Frequency	Percent
Parity		
1	277	28.0
2 to 3	481	48.6
≥ 4	231	23.4
Unintended pregnancy		
Yes	109	11.0
No	880	89.0
Abortion		
Yes	136	13.8
No	853	86.20
ANC during the index pregnancy		
Yes	817	82.6
No	172	17.4
Place of delivery		
Health Facility	808	81.7
Home	181	18.3
Postnatal care		
Yes	744	75.2
No	245	24.8
Time of Postnatal care visit		
Within the first week	280	37.6
2 to 6 weeks	341	45.8
> 6 weeks	123	16.5
Currently breastfeeding		
Yes	813	82.20
No	176	17.80

### Family planning methods used before the index pregnancy

**Fig. 1:**
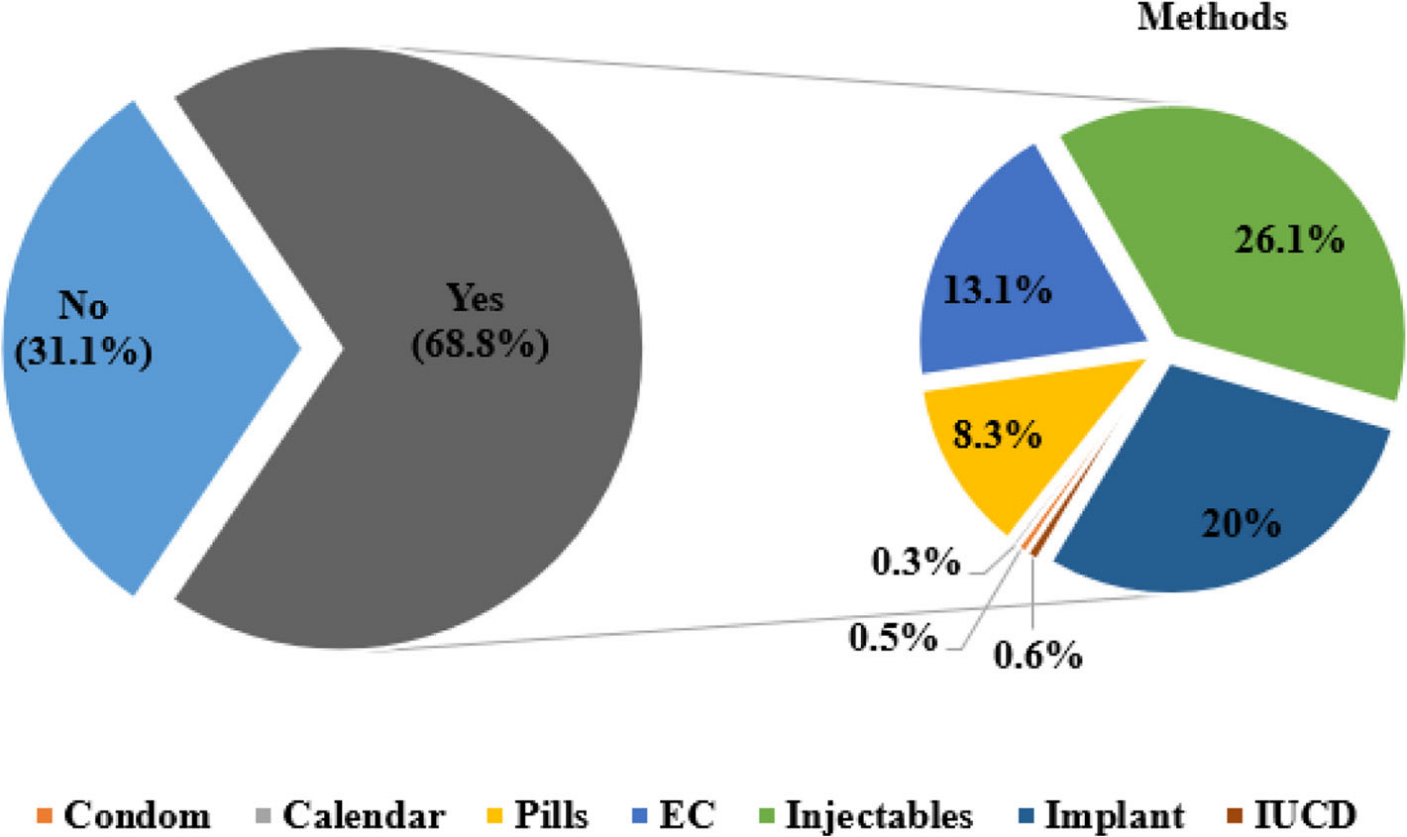
Family planning utilization before the index pregnancy among study participants, Western Ethiopia, 2020

The participants were asked whether they had used any methods of the family before index pregnancy and it showed that the majority, 68.80%, of postpartum mothers had used at least one method of family planning methods before the index pregnancy. Injectable forms and implants were the commonly used methods (Fig. 1**)**.

### Post-partum family planning use among study participants

**Fig. 2:**
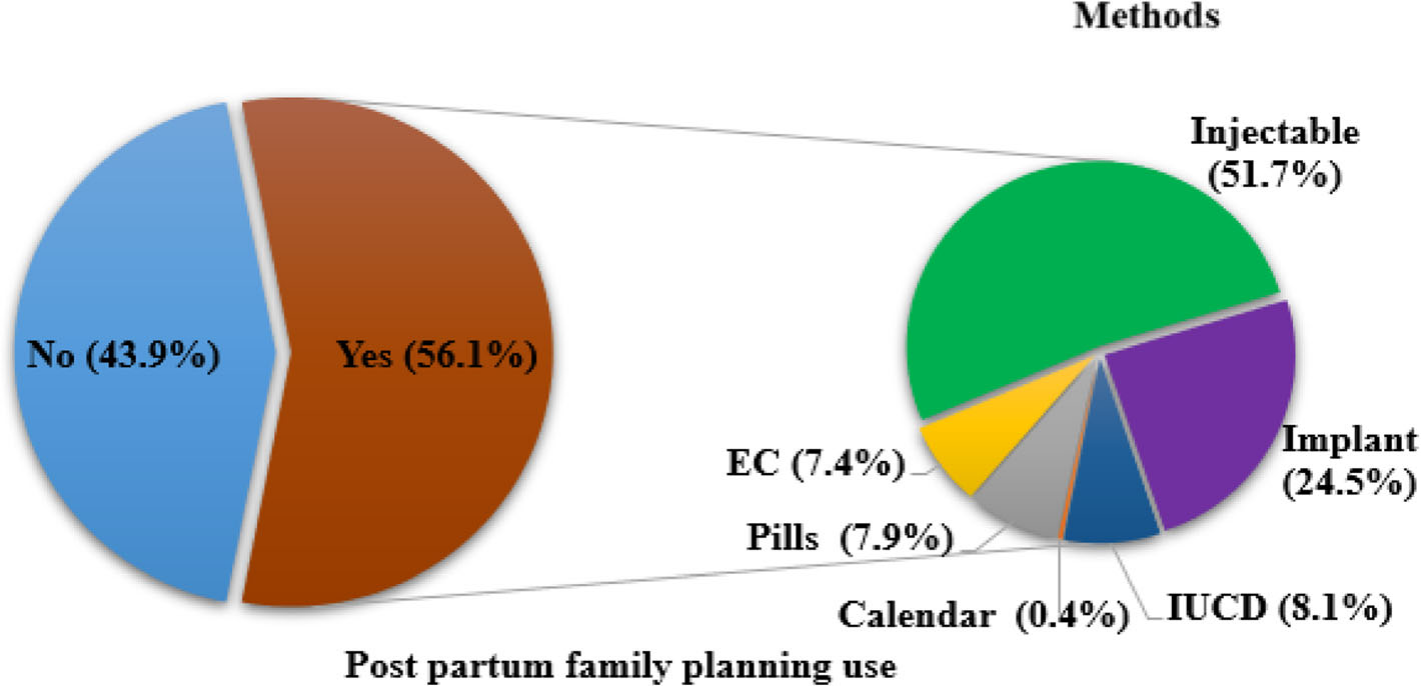
Postpartum family planning utilization among study participants, Western Ethiopia, 2020

Five hundred thirteen *(51.9%)* of the study participants didn’t discuss family planning with their husbands in the last year. However, more than half the participants *555 (56.1%)* had used family planning in the last year. The most common family planning methods used by participants were injectable form 287(51.7%) followed by implants (24.5%) (Fig. 2)*.*

Among those who used the postpartum family planning method majority, 267(48.1%) started the family planning method on the 45th day while those who started within a week and between six weeks to six months were 136(24.5%) and 91(16.4%) respectively. The main source of family planning method was health center 409(73.7%) followed by hospital 94(16.9%).

Four hundred one (72.3%) of postpartum family planning users reported as they were using the method they preferred. The foremost mentioned reasons for preference of the methods were a convenience for use 172(40.6%) and being comfortable to health 180(42.5%). On the other hand, the reasons mentioned by those participants who were using the non-preferred method were medical condition 78(59.5%) and lack of the preferred method in the health facility 58(40.5%).

Participants were asked if they did face difficulty accessing the family planning service during COVID-19 pandemics and one hundred fifty-seven (15.9%) responded as they did. The most common stated difficulties we’re unable to get transportation 100(63.7%) and absence of health extension workers 68(43.31%) while fear of acquiring coronavirus accounts for 19(12.1%) (Table 3).

**Table 3:** PPFP uptake among study participants, Western Ethiopia, 2020

*Characteristics*		*Frequency*	*Percentage*
Received counseling on PPFP during ANC and delivery	*Yes*	753	76.1
	*No*	236	23.9
Previous history of PPFP uptake	*Yes*	342	48.2
	*No*	368	51.8
Currently using PPFP	*Yes*	555	56.1
	*No*	434	43.9
Time of initiation of PPFP uptake	*Within 24 h*	30	5.4
	*Within a week*	136	24.5
	*2 to 6 weeks*	267	48.1
	*6 weeks to 6 months*	91	16.4
	*After 6 months*	21	5.5
Source of family planning	*Hospital*	94	16.9
	*Health center*	409	73.7
	*Health post*	33	5.9
	*Private clinic*	16	2.9
	*Pharmacy*	3	0.5
Availability of the preferred method	*Yes*	424	76.4
	*No*	131	23.6
Reason for preference of the method	*Convenient to use*	172	40.6
	*No need to remember*	33	7.8
	*Advised by friends*	39	9.2
	*Comfortable for health*	180	42.5
Reason for using non-preferred methods	*Medical reason*	78	59.5
	*Not available*	53	40.5
Faced difficulty during COVID-19 restrictions	*Yes*	345	34.8
	*No*	644	65.2

**Fig. 3:**
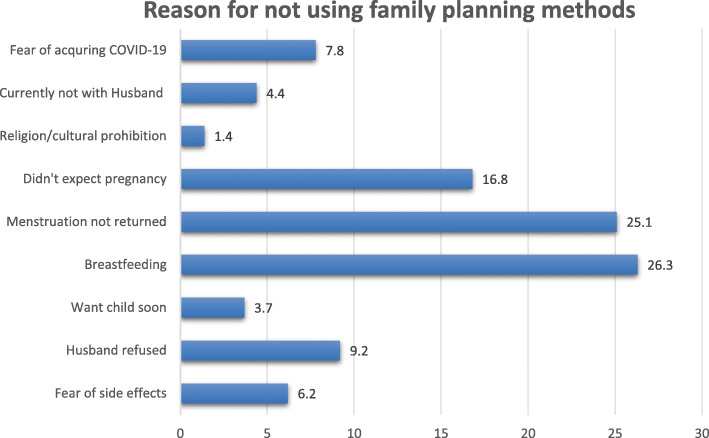
Reasons for not using family planning among study participants, Western Ethiopia, 2020

Moreover, 434(43.9%) of the study participants didn’t use any form of family planning method. A quarter of them mentioned the reasons for not using were breastfeeding 114(26.3%) and menses not yet resumed 109(25.1%) [Fig. 3].

### Factors affecting uptake of postpartum family planning service utilization

In bivariate analysis, utilization of postpartum family planning services was significantly associated with variables like great or equal to para two, history of using family planning before the index pregnancy, previous history of postpartum family planning utilization, counseling given during antenatal follow-up, and delivery on postpartum family planning utilization, place of delivery, a skilled birth attendant at birth, and ANC attendance for the index pregnancy. However, currently living with husband, history of abortion, history of unintended pregnancy, and COVID-19 restrictions have no positive association with the uptake of postpartum family planning services. After adjusting for confounding factors in the multivariate analysis, factors that significantly associated with utilization of postpartum family planning services were history of using family planning before the index pregnancy, previous history of postpartum family planning utilization, counseling during delivery on postpartum family planning utilization, place of delivery, a skilled birth attendant at birth and COVID-19 restrictions. The number of parties and ANC attendance for the index pregnancy were not significantly associated with the service utilization in this study.

Participants who had been counseled during antenatal care and delivery about postpartum family planning were 4.89 times more likely to use the service compared to their counterparts [AOR = 4.89 (2.31, 10.37)]. Postpartum mothers who gave birth at the health facility were more than 7,61 times likely to use the postpartum family planning service compared to those delivered at home [AOR = 7.61 (4.36, 13.28)]. It was also checked whether a skilled birth attendant is associated with the service utilization and the multivariable logistic regression result indicated that those mothers who were assisted by skilled birth attendants at birth were around 4.99 times more likely to uptake the service compare to those assisted by non-skilled attendants [AOR = 4.99 (2.88, 8.64)]. Postpartum family planning utilization was also 2 times higher among mothers who had used family planning before the index pregnancy [AOR = 2.09 (1.29, 3.41)].

COVID-19 restrictions were checked whether it affected uptake of the service and the result indicated that those postpartum mothers who faced restrictions of COVID-19 were by 39.5% less likely to use postpartum family planning services compared to those who did face restrictions [AOR = 0.59 (0.39, 0.90)] **(**Table 4**).**

**Table 4:** Multivariable Logistic Regression of factors affecting PPFP uptake among study participants, Western Ethiopia, 2020

Variables	PPFP utilization		COR at 95% C. I	AOR at 95% C. I	*P*-value
	Yes	No			
Parity					
1	219 (79.1%)	58 (20.9%)	0.24 (0.17, 0.34)	1.21 (0.80, 1.82)	0.36
2 to 3	231 (48%)	250 (52%)	0.22(0.15, 0.33)	1.18(0.71,1.73)	
≥ 4	105 (45.5%)	126 (54.5%)	1		
Family planning utilization before the index Pregnancy					
Yes	491 (68.5%)	226 (31.5%)	7.06 (5.12, 9.74)	2.09 (1.29, 3.41)	0.03*
No	64 (23.5%)	208 (76.5%)	1		
Previous history of PPFP utilization					
Yes	277 (51.0%)	266 (49.0%)	1.90 (1.33, 2.73)	1.07 (0.55, 2.07)	0.89
No	59 (35.3%)	108 (64.7%)	1		
Counseling on PPFP during ANC & delivery					
Yes	447 (59.4%)	306 (40.6%)	1.73 (1.29, 2.32)	4.89 (2.31, 10.36)	0.00*
No	108 (45.8%)	128 (54.2%)	1		
Place of delivery					
Health facility	525 (65.0%)	283 (35.0%)	9.34 (6.15, 14.18)	7.61 (4.35, 13.28)	0.00*
Home	30 (16.6%)	151 (83.4%)	1		
Skilled birth attendance					
Yes	450 (53.5%)	391 (46.5%)	**2.12 (1.45, 3.10)**	**4.99 (2.87, 8.63)**	0.00*
No	105 (70.9%)	43 (29.1%)	1		
Faced difficulty during COVID-19 restrictions					
Yes	185 (53.6%)	160 (46.4%)	**1.17 (0.9, 1.52)**	**0.59 (0.39, 0.9)**	0.01*
No	370 (57.5%)	274 (42.5%)	1		
ANC during the index pregnancy					
Yes	430 (58.8%)	301 (41.2%)	**1.52 (1.14, 2.02)**	1.03 (0.65, 1.65)	0.89
No	125 (48.4%)	133 (51.6%)	1		

*statistically significant

## Discussion

This study is aimed to assess barriers and determinants of postpartum family planning uptake among postpartum women in western Ethiopia. The study identified numerous factors that affect its uptake among postpartum women in western Ethiopia.

Nearly all of the study participants, (98.5%), had heard of at least one method of family planning. This is similar to studies conducted in Hossana town, Southern Ethiopia (98.6%) [12], and Aroressa (99.7%) [13]. However, the finding of the current study is higher compared to the study conducted in Bahirdar town, Northern Ethiopia [11]. This discrepancy could have resulted from differences in the study setting and period.

In the current study, despite the high level of awareness, the uptake of postpartum family planning was 56.1%. This finding is higher than studies conducted in Pakistan (24.6%) [26], Nigeria (40.6%) [14], Liberia(11.9%) [15], Uganda (28%) [16], and Aroressa district in Southern Ethiopia (31.7%) [13]. However, postpartum family planning uptake in this study is lower than studies conducted in Kenya (78.4%) [17], Debre Tabor town in Ethiopia (63%) [18], and Hosanna town in Ethiopia (73.9%) [12]. The low uptake in the current study might be explained by perceptions of low pregnancy risk due to breastfeeding and postpartum amenorrhea.

In this study, the most common type of postpartum family planning used by participants was injectable (51.7%) followed by Implants (24.5%]. This finding is similar to different studies conducted in Aroressa district [13], Hossana town [12], Debretabor town [18], and the Tigray region [34] in Ethiopia. This might be due to the similarity of the socio-cultural status of the community and user preferences. However, studies conducted in Pakistan [28], China [29], and Nigeria [21] showed condoms as the preferred method among study participants. The difference might be due to the study setting, awareness of the women about specific methods, and method mix strategies that different countries follow.

In this study, 72.3% of study participants were using the method they preferred. The most preferred method of contraceptive was injectable (64.39%.). However, because of the lack of the preferred method and medical contraindications, only 72.3% of study participants were using the method they preferred. In Iran, the most preferred method of family planning was minipills (29.3%) [19]. the study conducted in Turkey [35%] [20] and Rwanda (52%) [21], showed LARC methods as the most preferred method. The difference could be due to differences in the study setting and the strategies different countries are following.

In this study, 45.9% of the study participant did not start using any form of family planning methods. The major reasons mentioned were being breastfeeding (26.3%) and failure of menses to resume 109(25.1%). This could lead postpartum women to unintended pregnancies, abortions, and unplanned births [22]. Poor male engagement could also contribute to low utilization. Postpartum women experience amenorrhea for varying lengths of time, and their fertility can return before menses resume, even when breastfeeding [2]. Therefore, health professionals better deal with these misconceptions during counseling. Other studies in other countries identified different reasons. For instance, desire for more children and husband objection, and post-partum abstinence followed by fear of side effects of the methods and being breastfeeding were the dominant reasons in Turkey [20] and Liberia [15]. These differences might be explained by differences in the study setting, cultural differences, and health policies.

In the current study, participants who used family planning before the index pregnancy were twice more likely to use postpartum family planning than their counterparts. This is similar to the conducted in Addis Ababa where women who had no history of contraceptive use before their last birth were 88% less likely to use the family planning method during the postpartum period compared to those who had [23]. Therefore, promoting family planning utilization before pregnancy is essential to improve postpartum utilization.

Participants who received counseling on postpartum family planning during antenatal follow-up and delivery were five times more likely to utilize the services compared to those with not counseled. This is similar to the study conducted in Southern Ethiopia [24]. Therefore, strengthening counseling on postpartum family planning is important to promote its uptake.

This study showed that those participants who were assisted by skilled birth attendants at birth were 7 times more likely to utilize postpartum family planning compare to those assisted by non-skilled attendants. This is similar to the study conducted in Burundi and Rwanda where skilled birth attendance significantly affected the utilization of PPFP [16]. This could be because of the counseling health providers do during delivery.

The current study is conducted in the era of the COVID-19 pandemic where there are restrictions. The result indicated that those participants who faced difficulties during the COVID-19 pandemic were 39% less likely to use postpartum family planning services compared to those who did not face it. This is in line with the projection that some 47 million women in 114 low- and middle-income countries are to be unable to use modern contraceptives if the average lockdown, or COVID-19-related disruption, continues for 6 months with major disruptions to services [25]. The authors recommended the health sector design an effective option to maintain postpartum family planning services during COVID-19 restrictions or related health crises.

In this study, it is revealed that there is no association between ANC attendance and uptake of postpartum family planning. This is consistent with another finding in which no difference in PPFP use was observed between women who received PPFP counseling only in ANC and women who did not receive counseling at all [24].

### Strengths and limitations of this study

This study is the first of its kind invoving two regions, Oromia and Benshangul Gumuz regions, in western Ethiopia. The relatively large sample size and high response rate could make the findings generalisable to postpartum women in western Ethiopia. However, it was not without limitations. First, given the cross-sectional design of the study, it was not possible to establish cause-effect relationships of postpartum family planning uptake with independent variables. Second, partners (husbands) and health care provider perspectives were not addressed.

## Conclusion and recommendations

Post-partum family planning utilization among study participants was low. Given the associated factors, it is recommended that health facilities should make postpartum family planning one of their top priorities and focus on these factors to improve its utilization.

## Data Availability

The data sets used and analyzed during the current study are available from the corresponding author on formal request.

## Abbreviations

ANC: Antenatal care
AOR: adjusted odds ratio
COVID-19: corona virus disease 19
HSDP: Health Sector Development Plan
IUCD: Intrauterine Contraceptive Device
LAM: lactational amenorrhea
LMICs: low and middle-income countries
OR: odds ratio
PNC: postnatal care
PPFP: Postpartum Family Planning
WHO: World Health Organization
